# ‘Nanodentistry’: Exploring the beauty of miniature

**DOI:** 10.4317/jced.50720

**Published:** 2012-04-01

**Authors:** Rita Chandki, M. Kala, Kiran N. Kumar, Biji Brigit, Priyank Banthia, Ruchi Banthia

**Affiliations:** 1 M.D.S., Post graduate student. Department of conservative dentistry and Endodontics. Govt.Dental college and Research Institute, Bangalore, India; 2 M.D.S., Professor and Head. Department of conservative dentistry and Endodontics. Govt.Dental college and Research Institute, Bangalore, India; 3 M.D.S. Reader. Department of conservative dentistry and Endodontics, Govt.Dental college and Research Institute, Bangalore, Karnataka, India; 4 M.D.S. Senior lecturer. Department of conservative dentistry and Endodontics, Govt.Dental college and Research Institute, Bangalore, Karnataka, India; 5 M.D.S., Professor and Head. Department of Periodontics. Indraprastha dental college, Ghaziabad,India; 6 M.D.S., Professor. Department of Periodontics. Modern dental college and research center, Indore, India

## Abstract

Feynman’s early vision in 1959 gave birth to the concept of nanotechnology. He saw it as an unavoidable development in the progress of science and said that there is plenty of room at the bottom. Since then, nanotechnology has been part of mainstream scientific theory with potential medical and dental applications. Numerous theoretical predictions have been made based on the potential applications of nanotechnology in dentistry, with varying levels of optimism. While a few layers of nanotechnologic capability have become a reality for oral health in the last decade, many of these applications are still in their puerile stage .The most substantial contribution of nanotechnology to dentistry till date, is the more enhanced restoration of tooth structure with nanocomposites. The field of nanotechnology has tremendous potential, which if harnessed efficiently, can bring out significant benefits to the human society such as improved health, better use of natural resources, and reduced environmental pollution. The future holds in store an era of dentistry in which every procedure will be performed using equipments and devices based on nanotechnology. This article reviews the current status and the potential clinical applications of nanotechnology in dentistry.

** Key words:**Nanotechnology, nanodentistry, nanocomposites.

## Introduction

Last decade witnessed an unparalleled growth in all the fields of research in medicine. But there is little doubt that nanotechnology has the potential to change all aspects of medicine, health care and human life more profoundly than many other developments of the past taken together. Nanotechnology has revolutionized all aspects of health care into a new paradigm of state-of-the-art patient care beyond traditional, and dentistry is no exception.

The conceptual underpinnings of nanotechnologies were first laid out in 1959 by the noble prize winning physicist Richard Feynman in his lecture, “There’s plenty of room at the bottom”. In his historic lecture, he concluded saying, “this is a development which I think cannot be avoided”. (1) Since then, nanotechnology has come a long way to find its application in Supramolecular chemistry-Self assembling drug carriers and gene delivery systems, Nanoparticles and nanocapsules, Antibody technologies, Polymer-drug conjugates, Polymer-protein and antibody conjugates, Nano-precipitation, nanocrystals, Emulsification technologies, Liposome technology, In situ polymerization, Tissue engineering and repair, Dendrimer technologies ,Molecular imprinting including recent innovations in dental diagnostics, material and therapeutics. It has been proposed that nanodentistry will make it possible to maintain near-perfect oral health through the use of nanomaterials, (2-3) biotechnology (4-7) and nanorobotics. Referring to the two sides of the coin, as with all technologies, Nanotechnology possesses tremendous potential, but social issues of public acceptance, ethics, regulation, and human safety must be addressed before nanotechnology can be seen as the possibility of providing high quality dental care because nanotechnology carries a significant potential for misuse and abuse on a scale and scope never seen before.

This purpose of this article is to review current status of nanotechnology in dentistry and to provide an insight into what the future holds, highlighting the ethical and safety concerns associated with the use of nanotechnology.

## Approaches to nanodentistry

- Bottom-up approaches

- Top-down approaches

Bottom-up approaches (8)

- To arrange smaller components into more complex assemblies.

- DNA Nanotechnology utilizes the specificity of Watson-Crick base pairing to construct well-defined structures out of DNA and other nucleic acids.

Top-down approaches (9)

- To create smaller devices by using larger ones to direct their assembly.

## Nanodentistry as bottom-up approach

- Inducing anesthesia

- Major Tooth Repair

- Hypersensitivity Cure

- Dental Durability and Cosmetics

- Nanorobotic Dentifrice (dentifrobots)

- Tooth repositioning

- Local drug delivery 

- Nanodiagnostics

- Therapeutic aid in oral diseases.

Local Nanoanaesthesia

Dental treatment often involves injection of local anesthetic, which in turn comes with its longer duration of action and varying degrees of efficacy, patient discomfort and complications. Well-known alternatives, such as transcutaneous electronic nerve stimulation (TENS), cell demodulated electronic targeted anesthesia and other transmucosal, intraosseous or topical techniques, have proved to be of limited clinical efficacy.

Ongoing research to induce local anesthesia in the era of nanodentistry, is working on colloidal suspension containing millions of active analgesic dental nanorobotic particles that could be instilled on the patient’s gingivae. These nanorobots, after contacting the surface of the crown or mucosa, reach the dentin by migrating into the gingival sulcus and pass painlessly to the target site. On reaching the dentin, the nanorobots enter dentinal tubule holes that are 1 to 4 Am in diameter (10-12) and proceed toward the pulp, guided by a combination of chemical gradients, temperature differentials, and even positional navigation, all under the control of the onboard nanocomputer as directed by the dentist. Once installed in the pulp, the analgesic dental robots may be commanded by the dentist to shut down all sensitivity in any particular tooth that requires treatment. After completion of the treatment procedure, the dentist orders the nanorobots to restore all sensation, to relinquish control of nerve traffic and to egress from the tooth by similar pathways used for ingress.

Major tooth repair

Nanodental techniques for major tooth repair may evolve through several stages of technological development, first using genetic engineering, tissue engineering and tissue regeneration, and later involving the growth of whole new teeth in vitro and their installation. Ultimately, the nanorobotic manufacture and installation of a biologically autologous whole-replacement tooth that includes both mineral and cellular components— that is, complete dentition replacement therapy—should become feasible within the time and economic constraints of a typical office visit, through the use of an affordable desktop manufacturing facility, which would fabricate the new tooth, in the dentist’s office. Nanodentistry could also play a vital role in natural tooth maintenance.(13)

Tooth Repositioning

Orthodontic nanorobots could directly manipulate the periodontal tissues, including gingivae, periodontal ligament, cementum and alveolar bone, allowing rapid and painless tooth straightening, rotating and vertical repositioning within minutes to hours. (14) This offers an advantage over molar uprighting techniques currently in use, which require weeks or months to complete.

Dentin Hypersensitivity

Another pathological phenomenon that may be benefited by nanodental treatment is dentin hypersensitivity. (1) Dentin hypersensitivity is a common condition of transient tooth pain associated with a variety of exogenous stimuli. There is substantial variation in the response to such stimuli from one person to another. Except for sensitivity associated with tooth bleaching or other tooth pathology, the clinical cause of dentin hypersensitivity is exposed dentinal tubules as a result of gingival recession and subsequent loss of cementum on root surfaces (15-17).Reconstructive dental nanorobots could selectively and precisely occlude specific tubules within minutes, offering patients a quick and permanent cure. As nanorobots pass through the journey of enamel, dentin, reach into the pulp. Once installed in the pulp, having established control over nerve impulse traffic, the analgesic dental nanorobots may be commanded by the dentist to shutdown all sensitivity in selected tooth that requires treatment. When the dentist passes the icon for the desired tooth on the hand held controlled display monitor, the nerve is immediately anesthetized. After the oral procedures are completed, the dentist orders the nanorobots via the same acoustic data links to restore all sensation, to relinquish control the nerve traffic and to retrieve from the tooth via similar path. This analgesic technique is patient friendly as it reduces anxiety, needle phobia, and most important one is quick and completely reversible action. (1,18,19)

Durability and Appearance

The appearance and durability of teeth may be improved by replacing upper enamel layers with covalently bonded artificial materials such as sapphire (20) or diamond, which have 20 to 100 times the hardness and strength of natural enamel. Pure sapphire and diamond are brittle and prone to fracture, can be made more fracture resistant as part of a nanostructured composite material that possibly includes embedded carbon nanotubes. (21)

Nanorobotic dentifrice (Dentifrobots)

A subocclusal-dwelling nanorobotic dentifrice delivered by mouthwash or toothpaste could patrol all supragingival and subgingival surfaces at least once a day, metabolizing trapped organic matter into harmless and odorless vapors and performing continuous calculus debridement.(1)

Nanodiagnostics

Nanotechnologies already afford the possibility of intracellular imaging through attachment of quantum dots (QDs) or synthetic chromophores to selected molecules, for example proteins, or by the incorporation of naturally occurring fluorescent proteins that, with optical techniques such as confocal microscopy and correlation imaging, allow intracellular biochemical processes to be investigated directly.(22-24)

Even though microarray/ biochip methods making use of the detection of specific biomolecular interactions are now an indispensable tool for molecular diagnostics, there are some limitations. Nanotechnology is being applied to overcome some of the limitations of biochip technology. (25-26)

Therapeutic aid in oral diseases

Drug delivery 

Nanotechnology is opening new therapeutic opportunities for many agents that cannot be used effectively as conventional oral formulations because of their poor bioavailability. In some cases, reformulation of a drug with smaller particle size may improve oral bioavailability.(27) Nanoparticles formulations provide protection for agents susceptible to degradation or denaturation in regions of harsh pH, and also prolong the duration of exposure of a drug by increasing retention of the formulation through bioadhesion .Ideally, all these systems would improve the stability, absorption, and therapeutic concentration of the drug within the target tissue, as well as permit reproducible and long-term release of the drug at the target site.

Gene Therapy

Gene therapy is a recently introduced method for treatment or prevention of genetic disorders by correcting defective genes responsible for disease development based on the delivery of repaired genes or the replacement of incorrect ones.(28)Three main types of gene delivery systems have been described: viral vectors, nonviral vectors (in the form of particles such as nanoparticles, liposomes, or dendrimers), and the direct injection of genetic materials into tissues using so-called gene guns.(28,29) Applications of nanotechnological tools in human gene therapy has been reviewed widely by Davis, who described nonviral vectors based on nanoparticles (usually 50-500 nm in size) that were already tested to transport plasmid DNA. He emphasized that nanotechnology in gene therapy would be applied to replace the currently used viral vectors by potentially less immunogenic nanosize gene carriers. So delivery of repaired genes or the replacements of incorrect genes are fields in which nanoscale objects could be introduced successfully. (30)

## Nanodentistry as top down approach 

 (31)

- Nanocomposites 

- Nano Light-Curing Glass Ionomer Restorative

- Nano Impression Materials

- Nano-Composite Denture Teeth

- Nanosolutions 

- Nanoencapsulation 

- Plasma Laser application 

- Prosthetic Implants

- Nanoneedles 

- Bone replacement materials

Nanocomposites:

Currently, nanotechnology has had its greatest impact on restorative dentistry by offering refinements to already clinically proven resin based composite systems. Nanohybrid and nanofilled resin-based composites are generally the two types of composite restorative materials referred to under the term “nanocomposite”, usually in a context of particle size. Characterized by filler-particle sizes of ≤100 nm, these materials can offer esthetic and strength advantages over conventional microfilled and hybrid resin-based composite systems, primarily in terms of smoothness, polishability and precision of shade characterization, plus flexural strength and microhardness similar to those of the better-performing posterior resin-based composites. Beun and colleagues compared the physical properties of nanofilled, universal hybrid and microfilled composites, and observed a higher elastic modulus with the nanofilled RBC than most of the hybrids tested.(32)

Advantages:

- Superior hardness.

- Superior Flexural strength, modulus of elasticity and translucency.

- Reduced filling shrinkage.

- Excellent handling properties.

Nano Light-curing glass ionomer restorative

Blends Nanotechnology originally developed for Filtek™ Supreme Universal Restorative with fluoraluminosilicate (FAS) technology.

Advantages:

1. Superb polish.

2. Excellent esthetics.

3. Improved wear resistance

Clinical Indications:

- Primary teeth restorations.

- Transitional restorations.

- Small Class I restorations.

- Sandwich restorations.

- Class III and V restorations.

- Core buildups.

Impression Materials

Nanofillers are integrated in vinylpolysiloxanes, producing a unique addition of siloxane impression materials. The material has better flow, improved hydrophilic properties and enhanced detail precision. Nanofillers are integrated in the vinylsiloxanes, producing a unique addition siloxane impression material that offers better flow, improved hydrophilic properties, hence fewer voids at margin and better model pouring, enhanced detail precision.(33)

Advantages:

1. Increased fluidity

2. High tear resistance,

3. Hydrophilic properties

4. Resistance to distortion and heat resistance 

5. Snap set that consequently reduces errors caused by micro movements 

Nano-composite denture teeth 

Wear resistance is the most important physical properties of denture teeth .Porcelain denture teeth are most wear resistant, but they are brittle, lack bonding to the denture base, and difficult to polish. Acrylic resin denture teeth are easier to recontour, but undergo excessive wear.(31) Nanocomposite denture teeth comprises of Polymethylmethacrylate (PMMA), and uniformly dispersed nano - sized filler particles.

Advantages:

- Highly polishable, stain and impact resistant material 

- Lively surface structure 

- Superior surface hardness and wear resistance

Nanosolution

Nanosolutions produce unique and dispersible nanoparticles, which can be used in bonding agents. This ensures homogeneity and ensures that the adhesive is perfectly mixed everytime.(33)

Nanoencapsulation

SWRI (South West Research Institute) has developed targeted release systems that encompass nanocapsules including novel vaccines, antibiotics and drug delivery with reduced side effects. At present, targeted delivery of genes and drugs to human liver has been developed by Osaka University in Japan 2003. Engineered Hepatitis B virus envelope L particles were allowed to form hollow nanoparticles displaying a peptide that is indispensable for liver-specific entry by the virus in humans. Future specialized nanoparticles could be engineered to target oral tissues, including cells derived from the periodontium.

Laser Plasma Application for periodontia 

When TiO2 particle sizes are reduced to nanoscale (20-50 nm), and present on human skin in the form of a gel-like emulsion, it has some interesting properties such that when irradiated with laser pulses, these particles can be optically broken down with accompanying effects. (31)

- Shock wave

- Micro-abrasion hard tissue

- Stimulation of collagen production

Clinical applications:

1. Periodontal treatments 

2. Melanin removal 

3. Incision of soft tissue without anesthesia 

4. Caries preparation 

5. Cutting of enamel & dentin.

Prosthetic Implant 

Nanotechnologies may produce surfaces with controlled topography and chemistry that would help understan-ding biological interactions and developing novel implant surfaces with predictable tissue-integrative properties. Nanostructured surfaces may control the differentiation pathways into specific lineages and ultimately direct the nature of peri-implant tissues. Furthermore, it is possible to incorporate biologically active drugs such as antibiotics or growth factors during the precipitation of CaP coatings on Ti implants.(34,35) Compared with titanium alloy covered in micron-sized bumps, about 60% more new cells are grown on the same alloy containing nanometer-scale features, eg: Nanotite™ Nano-Coated Implant.

Nano Needles

Suture needles incorporating nano-sized stainless steel crystals have been developed. (Sandvik Bioloine, RK 91 TM needles (AB Sandvik, Sweden). Nanotweezers are also under development, which will make cell surgery possible in near future.

Bone replacement Materials

Chen et al. (36) took advantage of these latest developments in the area of nanotechnology to simulate the natural biomineralization process to create the hardest tissue in the human body, dental enamel, by using highly organized microarchitectural units of nanorod-like calcium hydroxyapatite crystals arranged roughly parallel to each other.

Hydroxyapatite nanoparticles used to treat bone defects are;

- Ostim ® (osartis GmbH Germany) HA.

- VITOSS ® (orthovita Inc, USA) HA+ TCP.

- NanOssTM (Angstrom Medica ,USA) HA

Barriers to overcome

- Precise positioning and assembly of molecular scale part.

- Economical nanorobot mass production technique.

- Simultaneous coordination of activities of large numbers of independent micron scale robots.

- Biocompatibility issue.

- Funding and strategic issues.

- Insufficient integration of clinical research.

- Inefficient translation of concept to product because of inadequate venture capital, excessive bureaucracy and lack of medical input

- Social issues of public acceptance, ethics, regulation and human safety.

## Conclusion

The aim of ‘Nanomedicine’ may be broadly defined as the comprehensive monitoring, control, construction, repair, defence and improvement of all human biological systems, working from the molecular level using engineered devices and nanostructures, ultimately to achieve medical benefit. Nanotechnology has a potential to transform the field of medicine, because it offers novel opportunities for sensing clinically relevant markers, molecular disease imaging, and tools for therapeutic intervention However, many challenges must be overcome if the application of Nanomedicine is to realise the improved understanding of the pathophysiological basis of disease, bring more sophisticated diagnostic opportunities, and yield more effective therapies and preventive measures.

Nanomedicine is clearly multidisciplinary and builds on the existing expertise in a large number of different scientific fields. Formal interdisciplinary training programmes should be instituted, focusing on basic scientific topics; for example molecular biology, colloidal chemistry, cell physiology, surface chemistry, and membrane biophysics towards a common goal of developing newer non invasive technologies that will benify oral health and general health at large. Nanotechnology is destined to become the core technology underlying all of 21st century medicine and dentistry.
